# Automated thorax disease diagnosis using multi-branch residual attention network

**DOI:** 10.1038/s41598-024-62813-6

**Published:** 2024-05-24

**Authors:** Dongfang Li, Hua Huo, Shupei Jiao, Xiaowei Sun, Shuya Chen

**Affiliations:** School of Information Engineering, Henan University of Science and Technology, Luoyang, 471000 Henan China

**Keywords:** Thoracic disease diagnosis, Convolutional neural network, Attention mechanism, BCE Loss, Label smoothing, Image processing, Machine learning, Predictive medicine

## Abstract

Chest X-ray (CXR) is an extensively utilized radiological modality for supporting the diagnosis of chest diseases. However, existing research approaches suffer from limitations in effectively integrating multi-scale CXR image features and are also hindered by imbalanced datasets. Therefore, there is a pressing need for further advancement in computer-aided diagnosis (CAD) of thoracic diseases. To tackle these challenges, we propose a multi-branch residual attention network (MBRANet) for thoracic disease diagnosis. MBRANet comprises three components. Firstly, to address the issue of inadequate extraction of spatial and positional information by the convolutional layer, a novel residual structure incorporating a coordinate attention (CA) module is proposed to extract features at multiple scales. Next, based on the concept of a Feature Pyramid Network (FPN), we perform multi-scale feature fusion in the following manner. Thirdly, we propose a novel Multi-Branch Feature Classifier (MFC) approach, which leverages the class-specific residual attention (CSRA) module for classification instead of relying solely on the fully connected layer. In addition, the designed BCEWithLabelSmoothing loss function improves the generalization ability and mitigates the problem of class imbalance by introducing a smoothing factor. We evaluated MBRANet on the ChestX-Ray14, CheXpert, MIMIC-CXR, and IU X-Ray datasets and achieved average AUCs of 0.841, 0.895, 0.805, and 0.745, respectively. Our method outperformed state-of-the-art baselines on these benchmark datasets.

## Introduction

The impact of thoracic diseases on global health and public welfare has been considerable over the years. Chest X-ray (CXR) images^1^ are currently the most common method that can effectively aid in the diagnosis of a series of thoracic diseases, such as cardiomegaly, consolidation, and edema. Computer-automated classification studies of multiple-label CXR images^2,3^ are extremely important in assisting clinical diagnosis. However, current publicly available CXR image datasets often contain several different diseases, and the location of lesions in these different diseases may overlap or interfere with each other. As a consequence, the diagnosis of thoracic diseases can be significantly affected. Currently, several methods^4–7^ for the automatic classification of multi-label CXR images have been proposed to improve the classification performance. However, studying computer-aided diagnosis (CAD) algorithms for thoracic diseases is still a challenging task. In recent years, many researchers have paid much attention to deep learning-based automatic medical image analysis techniques, and some techniques have been applied to medical image processing, such as U-net^8^, PSPNet^9^, DeepLab^10^ for medical image segmentation, ResNet^11^, DenseNet^12^ and Transforms^13^ for medical image classification. To learn case information from CXR images corresponding to chest diseases and to help physicians in clinical diagnosis, some methods have been used to improve the learning ability of network models. These methods can be broadly classified into three categories: (1) optimizing the network structure, (2) introducing attention mechanisms, (3) optimizing the loss function. In addition, The public release of two large datasets, ChestX-Ray14^2^ and CheXpert^3^, has further contributed to the research in this area.

The diagnostic steps of chest diseases include feature extraction of abnormal regions and disease classification. To improve the ability of the model for feature extraction, the main approach is to optimize the structure of the network model. For example, Wang et al.^2^ used a transition layer, a global pooling layer, and a prediction layer after the last convolutional layer instead of the fully connected layer and the final classification layer in DCNN. This method is able to find the reasonable spatial location of the disease. Chowdary et al.^14^ designed a two-branch network that firstly segmented the input CXR image by R-I UNet, and then used two fine-tuned AlexNet models to extract features from the original CXR image and the segmented image respectively. Hashmi et al.^15^ fine-tuned five classical CNN models using transfer learning and achieved good improvements in CXR classification. Chen et al.^6^ proposed a DualCheXNet to predict thoracic diseases, using DenseNet and ResNet to extract features from the same CXR image, and then using two auxiliary classifiers to classify the features. Huang et al.^16^ proposed an HRNet to extract abnormal features from four feature maps with different resolutions and classify them. With the advancement of attention mechanisms and their applications for image classification tasks in recent years, the performance of

**Figure 1:**
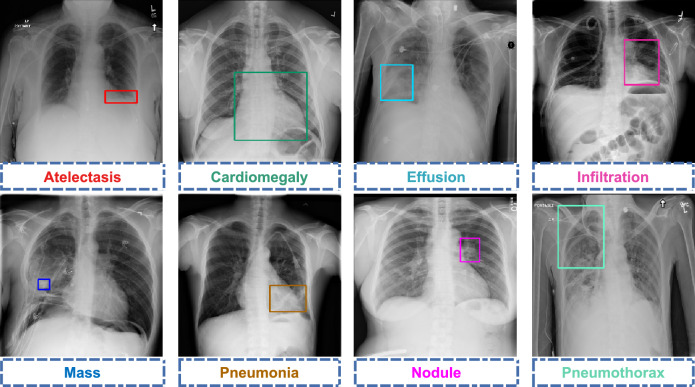
Examples of some of the lesion areas on the ChestX-Ray14 dataset. Each thoracic disease appears in a different lesion area. The disease present in the bounding box on each CXR image corresponds to the name of the pathology of the same color below the image.

network models have been further improved. For example, the ECA^17^ attention mechanism used in MRFCNet^18^ can adaptively calibrate the channel response of the feature maps and enhance the main pathological features while suppressing the transmission of useless information. Meanwhile, many other attention mechanisms such as SE^19^, CBAM^20^, and CA^21^ have been proposed and applied in many studies. Inspired by^18^, in our study, after comparing the effects, we finally introduced CA into feature extractor to help our network locate the abnormal regions on CXR images.

However, in many research works, the spatial location and channel information on various diseases tend to be disregarded, resulting in models that fail to adequately capture details regarding lesion regions across different scales. Furthermore, a prevalent issue of class imbalance is evident in the majority of datasets, which can easily give rise to overfitting challenges. Regrettably, existing research methodologies lack efficient strategies to address these issues, significantly impacting the ultimate classification outcomes. Additionally, as depicted in Fig. 1, a substantial amount of irrelevant data concerning non-diagnostic regions is present in chest X-ray (CXR) images. This not only incurs heightened computational expenses but also adversely affects the final classification performance of the model. Although the approaches proposed in^22,23^ aim to localize regions of interest and integrate them with global images for chest radiograph classification, they do not effectively tackle the issue.

To solve these issues, we propose a multi-branch residual attention network (MBRANet) model, which focuses on the fusion and classification of image features at several different scales. MBRANet works on abnormal region extraction features by using ResNet50. With the emergence and application of attention mechanisms in recent years, we introduced the CA to focus attention on specific locations in the images. Subsequently, leveraging the premise of a Feature Pyramid Network (FPN), we conduct multi-scale feature fusion in an ensuing manner. Then, we designed a new multi-branch feature classifier (MFC) based on the class-specific residual attention (CSRA) method to classify the extracted multi-scale feature information. In addition, to solve the problem caused by class imbalances, we designed the BCEWithLabelSmoothing loss function. Finally, we use a heat map to display the anomalous regions on the CXR images. In summary, the main contributions of this work are as follows:

- We proposed the MBRANet model for multi-label CXR image classification. The model can better capture the location information of disease-correlated regions in CXR images. Coordinate attention (CA) is proposed to make the resident blocks pay more attention to the disease-correlated regions and retain more discriminative features. Enhancing feature fusion through the integration of the Feature Pyramid Network (FPN) approach.
- We proposed a novel Multi-branch Feature Classifier (MFC) that effectively classifies features at multiple scales. This is achieved by incorporating the class-specific residual attention (CSRA) module, which eliminates the need for parameterization during the classification process. Our method overcomes the limitation of the fully connected layer in utilizing spatial information from features.
- We designed a novel BCEWithLabelSmoothing loss function, which incorporates a smoothing factor. The inclusion of this factor addresses the issue of model sensitivity to noisy or uncertain data when utilizing traditional one-hot encoding for labels.
- We developed a graphical user interface (GUI) page for visualizing the lesion area and displaying classification results. Experimental evaluations conducted on extensive CXR image datasets consistently demonstrate that our proposed MBRANet surpasses previous competing approaches in terms of performance, thus confirming the efficiency of our method.

The following sections of this paper are organized as follows: “Related work” introduces work on deep learning and attention mechanisms for multi-label CXR image classification. The “Approach” section describes the method used in the proposed MBRANet model. The “Experiment” section presents the dataset and experimental parameters (such as learning rate, and batch size), as well as a summary of the experimental results. The “Analysis and discussions” section encompasses a comprehensive evaluation of the results obtained from ablation experiments on each module, alongside presenting case studies. Furthermore, it delves into a thorough examination of the problem addressed in this paper, emphasizing the significance of the research while also recognizing the existing limitations inherent within our current methodology. Finally, the “Conclusion” section concludes our work.

## Related work

In this section, we summarize related research in three aspects: Firstly, we present some previous studies of work on the classification of multi-label chest X-rays. Secondly, we give an analysis of attention mechanisms. In conclusion, we examine some of the significant research currently available in this area.

### Multi-label chest X-ray image classification

In recent years, computer-aided diagnosis (CAD) research has attracted widespread interest and made significant breakthroughs. With the release of the publicly available ChestX-ray14, CheXpert, and COVID-19, more researchers are applying deep learning to automated CXR analysis. Specifically, the ChestX-ray14 dataset provided by the NIH Clinical Center has been a hot spot for research in automated CXR image analysis. Wang et al.^2^ used four pre-trained ImageNet^24^ models to evaluate the performance of multi-label classification of chest X-ray images and found that ResNet50 performed best. Currently, a basic approach is to classify CXR images by training a binary classifier for each lesion by using the popular CNN. Ma et al.^25^ proposed a ChestXNet model, an improvement on the pre-trained DenseNet121^12^, to classify each chest X-ray image for abnormalities and achieve excellent results in detecting pneumonia. Hashmi et al.^15^ used transfer learning to fine-tune five classical CNN models and proposed a weighted classifier that combined the classification results of these CNN models to achieve high accuracy in identifying pneumonia. Chen et al.^6^ used a dual asymmetric architecture based on ResNet and DenseNet to adaptively capture more discriminable features on thoracic CXR images. Kumar et al.^26^ proposed a PairWise Error (PWE) loss function and an optimized convolutional network for multi-label chest X-ray image classification. Albahli et al.^27^ proposed a new method called AI-CenterNet with DenseNet-41, which uses a Heatmap header to find the lesion region and its class, and then Multitask loss is used to further improve the localization and classification of the lesion region. Chen et al.^28^ introduced graph convolutional networks (GCN) for lung disease classifying. However, these methods use mainstream deep learning networks to extract pathological features from CXR images, which are not capable of combining global and local features. Therefore, we utilized the multi-scale features extracted by the network to learn more relevant information.

### Attention learning

The attention mechanisms enhance the model’s ability to handle lengthy sequences and intricate relationships. Conventional sequence models often fail to effectively capture long-range dependencies, whereas attention mechanisms overcome this limitation by attending to different positions of the input sequence. This dynamic adjustment of attention enables the model to capture both local and global information within the sequence.

The attention mechanisms are important in the classification of multi-label chest X-ray images. An attention mechanism can enhance model performance by enabling it to concentrate on the lesion region in the image. Guendel et al.^29^ proposed a location-aware approach. This method can effectively utilize high-resolution image data and significantly improve classification accuracy by incorporating spatial information about the disease. Moreover, they proposed a new split reference for the dataset, which can be meaningfully benchmarked for future directions. Wang et al.^30^ developed a triple attention network (A3Net). Specifically, A3Net uses the pre-trained DenseNet121 model as the backbone network for feature extraction and integrates three attention modules into a unified framework that learns attention to information at the channel level, element level, and scalar level. To further improve the performance of individual disease diagnosis, Guan et al.^31^ suggested exploring local discriminative regions and proposed attention-guided masked inference to help the network recognize diseases. Zhu et al.^32^ proposed a pixel classification and attention network (PCAN) for disease classification and weakly supervised localization, and they also provided interpretability for disease classification. Ouyang et al.^33^ proposed an attention-driven weakly supervised algorithm that can be used to solve the problem of anomaly localization. The algorithm contains a hierarchical attention-mining framework that unifies the visual attention of activation functions and gradients in a single whole. Chen et al.^34^ proposed a new network PCSANet that is based on pyramidal convolution and shuffle attention modules for thoracic disease classification and COVID-19 detection.

### Other works

In addition to the aforementioned research aspects, several other valuable contributions have been made in this field. For example, optimizing network structure is a crucial research direction. By fine-tuning the CNN model, a comprehensive feature representation is achieved. Baltruschat et al.^35^ optimized the ResNet-50 architecture, which better extracts information from images and improves its applicability to the classification of chest CXR images. Albahli et al.^36^ proposed a new strategy to complement three deep CNN models with synthetic data to identify 14 pulmonary-related conditions. This approach uses three algorithms: DenseNet121, ResNet152V2, and InceptionResNetV2. Besides, the proposed models have been trained and tested separately. Currently, many studies have classified different lung diseases in the ChestX-ray14 dataset. However, due to the class imbalance in this dataset, these models may be over-trained for diseases in one category and under-trained for diseases in another. As a result, although these models can successfully detect multiple regions of lung disease, their performance is ultimately inadequate when applied more generally. In many studies of imbalanced data, such as^37^ most choose to address the problem of class imbalance by employing data augmentation techniques on the dataset. Such as up-sampling operations for a small number of classes and down-sampling operations for a large number of classes. We have instead viewed the classification of each disease as a dichotomous problem (Yes or No), using the BCE Loss function and the Label smoothing technique to address the class imbalance.

**Figure 2:**
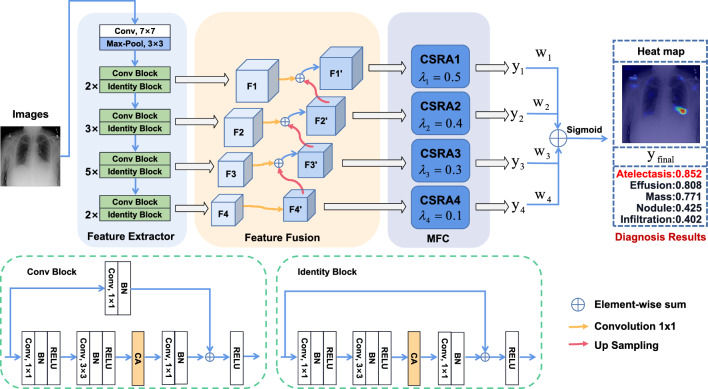
Framework of the proposed MBRANet model. The MBRANet architecture consists of a feature extractor, feature fusion, and a multi-branch classifier (MFC). Initially, the input CXR image is fed into the feature extractor, generating four distinct feature scales, labeled as *F*1, *F*2, *F*3, and *F*4. Subsequently, the feature fusion process is carried out using the FPN technique, resulting in the creation of new features denoted as $F1^{'}$, $F2^{'}$, $F3^{'}$, and $F4^{'}$. These fused features are then subjected to classification using the MFC approach, resulting in classified feature vectors namely $y_1$, $y_2$, $y_3$, and $y_4$. Finally, the heat map of the lesion area is obtained, as well as the final prediction $y_{final}$ using the weighted decision fusion method.

## Approach

In this section, we introduce the overall design of the MBRANet network model proposed in this paper. First, We propose incorporating a residual structure as a feature extractor and implementing attention mechanisms to enhance the feature extraction capability of the model. Next, we will describe the designed Multi-Branch classifier module in detail. Finally, we will introduce how the BCEWithLabelSmoothing loss function works. The diagram of our model is illustrated in Fig. 2. We now give further details of the MBRANet model.

### CNN-based feature extractor

Feature extraction is an important step in identifying and classifying abnormal regions in thoracic CXR images. Therefore, effective features are needed to locate the abnormal regions correctly. The attention mechanisms can further channel information and location information in the images. Feature fusion methods combine information from different levels. By integrating diverse features, the model gains a more comprehensive understanding of the data, resulting in better results for complex tasks.

**Backbone:** In CXR images, there are significant differences in lesion size among different diseases, as shown in Fig. 1. Therefore, the desired network model needs to have robust multi-scale feature extraction ability.

**Table 1:** Network structure of the feature extractor, where Conv k $\times$ k block stands for k $\times$ k convolution, Batch Normalization, and ReLU. CA represents the Coordinate Attention operation in Fig. 4c. F1, F2, F3, and F4 represent the output tensor of the different layers.

Layer	Output size	Network architecture	Feature map
Stem	112 $\times$ 112	Conv 7 $\times$ 7, Max pool 3 $\times$ 3	F0
Conv1_x	56 $\times$ 56	$$\left( \begin{array}{c} \text {Conv 1}\times \text {1} \\ \text {Conv 3}\times \text {3} \\ \text {CA} \\ \text {Conv 1}\times \text {1} \\ \end{array}\right) \times \text {3}$$	F1
Conv2_x	28 $\times$ 28	$$\left( \begin{array}{c} \text {Conv 1}\times \text {1} \\ \text {Conv 3}\times \text {3} \\ \text {CA} \\ \text {Conv 1}\times \text {1} \\ \end{array}\right) \times \text {4}$$	F2
Conv3_x	14 $\times$ 14	$$\left( \begin{array}{c} \text {Conv 1}\times \text {1} \\ \text {Conv 3}\times \text {3} \\ \text {CA} \\ \text {Conv 1}\times \text {1} \\ \end{array}\right) \times \text {6}$$	F3
Conv4_x	7 $\times$ 7	$$\left( \begin{array}{c} \text {Conv 1}\times \text {1} \\ \text {Conv 3}\times \text {3} \\ \text {CA} \\ \text {Conv 1}\times \text {1} \\ \end{array}\right) \times \text {3}$$	F4

We construct a feature extractor by employing residual blocks inspired by the ResNet50 network architecture, which can represent multi-scale features at a finer granularity. The relationship between the input and output of a bottleneck residual unit is shown in  (1).

1 $$\begin{aligned} {\text {H(x)}} = F(x) + x \end{aligned}$$

where x denotes the input of this residual unit; F(x) denotes the residual value of the input after the convolution layer; H(x) represents the output of the current residual unit.

**Figure 3:**
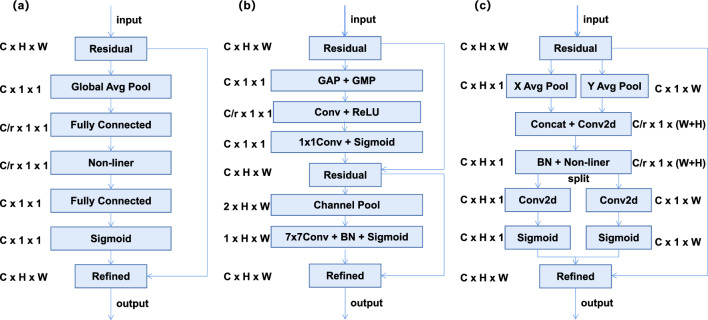
Schematic comparison of the proposed coordinate attention block(c) to the classic SE channel attention block^19^ (**a**) and CBAM^20^ (**b**). Here, “GAP” and “GMP” refer to the global average pooling and global max pooling, respectively. “X Avg Pool” and “Y Avg Pool” represent pooling operations in horizontal and vertical directions. “Split” will decompose the features obtained in the previous step.

**Coordinate attention (CA):** The convolution operation in residual blocks can capture local relations, but it cannot capture the dependency of long-range pixels. To solve this problem, we introduce the Coordinate Attention (CA) module into residual blocks, the main idea of the CA module is to calculate the attention weight of each position through a set of learnable parameters. The weights are utilized to emphasize significant input features, enabling the model to concentrate on important locations. The structure is shown in Fig. 3c. This module can effectively capture channel relationships and spatial location information in CXR images. Since the module has the same input and output dimensions, it can be flexibly inserted into any network structure to alleviate the problem of inadequate extraction of

local features by convolutional operations. We introduce CA into the residual block to obtain a new residual block for feature extraction.

**Output of feature extraction:** The feature extractor consists of multiple stages. As shown in Table 1, the network architecture consists of the following parts, a $7 \times 7$ convolutional layer in the stem, a $3 \times 3$ maximum pooling layer, and four consecutive stages containing different numbers of ResNet blocks. After a series of convolutional operations, we get the corresponding output feature maps *F*1, *F*2, *F*3, and *F*4 from $Conv1\_x$, $Conv2\_x$, $Conv3\_x$, and $Conv4\_x$.

**Figure 4:**
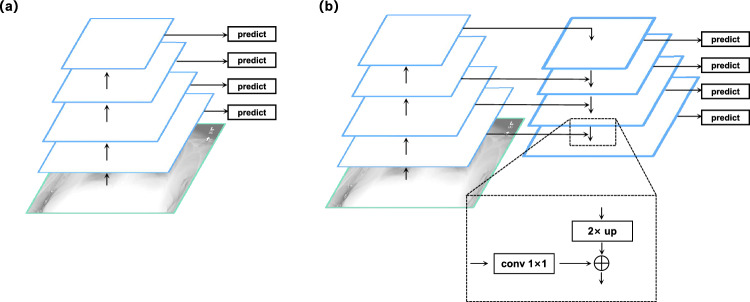
(**a**) Using an image to build a feature pyramid. (**b**) Feature Pyramid Network with top-down skip connection architecture and a building block that demonstrates the lateral connection and the top-down pathway, merged through addition.

**Feature fusion:** During the extraction and classification of multi-scale features, the lack of direct skip connections to leverage deep features from various scales (as depicted in Fig. 4a) leads to an inadequate capture of feature details and contextual information. To address this, we employ the feature fusion approach known as the Feature Pyramid Networks (FPN). As depicted in Fig. 4b, we first reduce the dimensions of *F*1, *F*2, *F*3, and *F*4 using 1x1 convolutions. We then adopt a top-down pathway propagation, starting from higher-level feature maps. This process involves retrieving feature maps from the previous layer and upsampling them using bilinear interpolation. The feature maps of the same scale are subsequently fused using element-wise addition, resulting in more refined feature maps denoted as $F1^{'}$, $F2^{'}$, $F3^{'}$, and $F4^{'}$. This fusion process effectively combines lower-resolution yet semantically rich features with higher-resolution features, resulting in an enhanced capture of feature details.

### Multi-branch feature classifier (MFC)

There may be several abnormal areas on a single chest X-ray. The type of lung disease is diagnosed based on these abnormal regions. We want the model to focus not just on the global information of the image, but also on the spatial location of the abnormal regions in the feature map, which can then help experts assist in the diagnosis of lung disease. To achieve this goal, we designed the MFC method, which allows our network model to focus more on the key locations of diseases in the CXR image, in order to reduce the focus on irrelevant information in the image.

**Figure 5:**
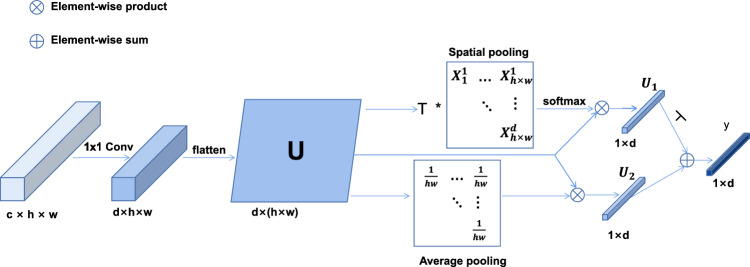
The proposed CSRA module to obtain features and classification results.

We designed the MFC method with four classifiers using the class-specific residual attention (CSRA) module. Zhu et al.^38^ proposed the Residual Attention module to efficiently capture the different spatial regions occupied by different classes of objects, which can make full use of the spatial attention of each object class and achieve higher accuracy on the task of multi-label classification. The working principle of the CSRA module is shown in Fig. 5.

As shown in  (2), for a given image *S*, it is first passed into the network model $\varphi$ for feature extraction to obtain the feature tensor $F \in R(c \times h \times w)$, where *c*, *h*, and *w* are the dimension, height, and width of the feature tensor, respectively. As shown in Table 1, the extracted features *F*1, *F*2, *F*3 and *F*4 have the shapes $256 \times 56 \times 56,512 \times 28 \times 28, 1024 \times 14 \times 14$ and $2048 \times 7 \times 7$ respectively. Then, we feed the extracted $F1$ $\sim$ $F4$ into the CSRA module to classify. The CSRA module first performs a $1 \times 1$ convolution operation on the input feature vector $c \times h \times w$ to reduce its dimensionality to $d \times h \times w$ and decouple it into $x_1$, $x_2$, $x_3, \ldots , x_{h \times w}$, where d denotes the number of classes. Afterward, the feature tensor after the dimension reduction

operation (Flatten) is transformed into a one-dimensional vector $U:d \times (h \times w)$ for the subsequent classification task. Next, two distinct operations are performed on the information tensor after the Flatten operation. First, a Spatial pooling operation is performed on *U* to obtain a spatial attention score map $U_1: 1 \times d$ describing the spatial feature information of a category. Then, the Average pooling operation is executed on *U* to obtain the classical global category diagnostic feature vector $U_2: 1 \times d$, As shown in Fig. 5. *y* = [$y^1$, $y^2,\ldots , y^c$], c means the number of classes. The predicted probability of the k-th class can be derived by the following (3).

2 $$\begin{aligned} {\text {F}}= & {} \varphi ({\text {S; }}\theta ) \end{aligned}$$

3 $$\begin{aligned} {{\text {y}}^k}= & {} \frac{1}{{hw}}\sum \limits _{i = 1}^{hw} {X_i^k} + \lambda \sum \limits _{i = 1}^{hw} {{\text {softmax(T}}X_i^k)} X_i^k \end{aligned}$$

The parameter $\theta$ refers to the setting of the parameter in the network model. T is the temperature hyperparameter (T > 0), which controls the sharpness of a single position score. $\lambda$ is a parameter that controls the output weight of spatial pooling. We initialized the parameter *t* with a value of 1. In our experiments, we feed $F1^{'}\sim F4^{'}$ into the classifier and set $\lambda$ to 0.5, 0.4, 0.3, and 0.1 respectively. Finally, the prediction vectors $y_1$, $y_2$, $y_3$, and $y_4$ output by four CSRA modules are added for a joint judgment of thoracic diseases: $y_{final} = \sigma (\sum \nolimits _{i=1}^{4}w_iy_i)$.

### Multi-label classification loss

These datasets have the problem of class imbalance. Such as in the distribution of the number of categories in the ChestX-Ray14 dataset, the number of positive images such as ‘Pneumonia’, ‘hernia’, and ‘Cardiomegaly’ is far less than the number of negative samples in the distribution of positive CXR images of the thorax. However, the unbalanced distribution of samples hinders the accuracy of classification with multiple labels and requires more pathological information to train a model with good results. To address this problem, we use Label Smoothing^39^ to optimize the Binary Cross-Entropy (BCE) loss. we have termed this the BCEWithLabelSmoothing loss function and used it in our MBRANet.

For classification problems, we typically assume that the training data’s label vector has a probability of 1 for the target category and 0 for the non-target category. The traditional label vector $y_i$ for computing one-hot encoding is shown in  (4). Cross-entropy loss is a commonly used loss function for binary classification problems, of which BCE (Binary Cross-Entropy) loss is a special case for binary classification tasks. The equation for BCE Loss is shown in  (5).

4 $$\begin{aligned} y_i = \left\{ \begin{array}{ll} 1,&{}\quad {\text {i}} = target\\ 0,&{}\quad {\text {i}} \ne target \end{array} \right. \end{aligned}$$

5 $$\begin{aligned} \begin{aligned} BCELoss = - (y \times \log ({\text {p(x)}}) + (1 - y) \times \log (1 - p(x))) \end{aligned} \end{aligned}$$

where *y* is the true binary label (0 or 1), and *p*(*x*) is the predicted probability value.

During training, label smoothing reduces the overconfidence of the model in the training sample by replacing the true label with a small value between 0 and 1. Label smoothing incorporates a uniform distribution and replaces the traditional one-hot encoded label vector $y_i$ with an updated label vector $\widehat{y_i}$. $\widehat{y_i}$ is calculated as shown in  (6).

6 $$\begin{aligned} \begin{aligned} \widehat{{\text {y}}_{\text {i}}} = \left\{ \begin{array}{ll} 1 - \alpha ,&{}\quad {{\text {y}}_{\text {i}}} = 1\\ \alpha {\text {/K}},&{}\quad {{\text {y}}_{\text {i}}} = 0 \end{array} \right. \end{aligned} \end{aligned}$$

Where K is the number of classes. $\alpha$ is the smoothing factor, which is usually taken as a small positive number (e.g. 0.1 or 0.01), used to control the degree of label smoothing. By introducing the smoothing factor, the degree of overconfidence of the model for the training samples can be reduced, thus improving the generalization ability and robustness of the model. BCEWithLabelSmoothing loss is calculated as shown in  (7).

7 $$\begin{aligned} \begin{aligned} BCEWithLabelSmoothing&= - \frac{1}{K}\sum \limits _{i = 1}^K (\widehat{{y_i}} \times \log ({p_i})\\&\quad + (1 - \widehat{{y_i}}) \times \log (1 - {p_i})) \end{aligned} \end{aligned}$$

where the label of each CXR image is labeled as a one-hot vector *y* = [$y_1$, $y_2,\ldots , y_K$]. In our conducted experiments, the value of *K* was determined to be 14, while the parameter $\alpha$ was deliberately configured to a fixed value of 0.1. The label $\widehat{y_i}$ is obtained after the calculation of the *y* label in  (6).

**Table 2:** Distribution of training, validation, and test sets in ChestX-Ray14, ChexPert, MIMIC-CXR, and IU X-Ray datasets.

Dataset	ChestX-Ray14	CheXpert	MIMIC-CXR	IU X-Ray
train	78,468	224,116	368,960	5226
val	11,219	200	2991	748
test	22,433	–	5159	1496

### Ethical and informed consent for data used

This article is licensed under the Creative Commons Attribution 4.0 International License. Based on the terms of this license, the Licensed Material can be copied and shared in whole or in part. Additionally, adapted material can be created, copied, and shared. This license is free, non-transferable, non-exclusive, and irrevocable, and applies worldwide. The hyperlink to access the licensed material is https://creativecommons.org/licenses/by/4.0/.

## Experiment

In this section, we will validate the performance of the MBRANet model proposed in this paper for multi-label CXR image classification on the ChestX-Ray14, CheXpert, MIMIC-CXR, and IU X-Ray datasets. First, we briefly overview the basic information about the experimental datasets. Then, we describe the implementation details of the experiments. Next, we present the evaluation metrics. Finally, comparisons are made between the performance of the MBRANet model and state-of-the-art methods.

### Dataset

**Figure 6:**
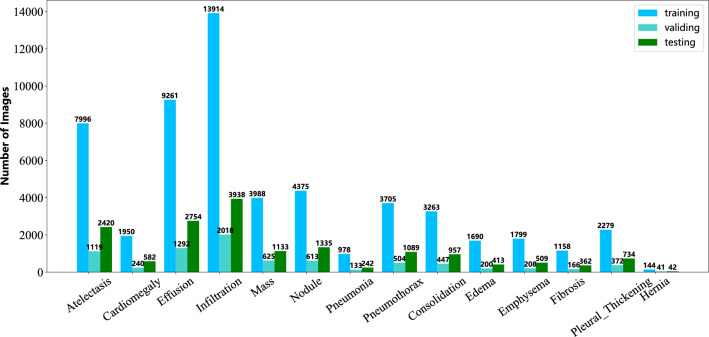
Distribution of training, validation, and testing images over 14 disease categories in the Chest X-ray14 dataset.

**ChestX-Ray14**^2^ is a publicly available dataset widely used for medical image analysis, which is primarily used for the automated detection and classification of chest diseases. The dataset is published by the National Institutes of Health (NIH) and covers 14 common categories of chest disease (atelectasis, cardiomegaly, effusion, infiltration, mass, nodule, pneumonia, pneumothorax. consolidation, edema, emphysema, fibrosis, pleural thickening, and hernia). The disease labels in each image are extracted from the relevant radiology reports using natural language processing. It contains 112,120 frontal X-ray images of size 1024 $\times$ 1024 obtained on 30,805 patients. Some examples of which are illustrated in Fig. 1. As shown in Table 2, the splitting of this dataset strictly followed the official splitting criteria published by^2^ (78,468 images (70%) for training, 11,219 images (10%) for verification, and 22,433 images (20%) for testing). The distribution of the number of images used for training, validation, and testing across all classes is shown in Fig. 6 and reflects the high imbalance and diversity of the ChestX-Ray14 dataset.

**CheXpert**^3^ is a large dataset of CXR images published by Stanford University. It consisted of 224,316 chest radiographs of 65,240 patients. Each report was labeled for the presence of 14 observations as positive(1), negative(0), or uncertain(−1). The frequently used methods for handling uncertain(−1) labels were U-Ones (replacing all uncertain labels with 1) and U-Zeroes (replacing all uncertain labels with 0). According to Table 2, we split the dataset according to the official proportions^3^, where the validation set consisted of 200 chest radiology studies, manually annotated by three certified radiologists.

**MIMIC-CXR**^40^ is a large publicly available dataset of chest radiographs with free-text radiology reports. The dataset contains 377,110 images corresponding to 227,835 radiographic studies performed at the Beth Israel Deaconess Medical Center (BIDMC) in Boston, MA. According to Table 2, we adopt the standard 7:1:2 train/validation/test splits to verify the generalization capability of the MBRANet model.

**IU X-Ray**^41^ is a public radiography dataset collected by Indiana University, containing 7470 chest X-ray images and 3955 radiology reports. As shown in Table 2 in the official documentation, the dataset is divided into training, validation, and test sets with varying proportions of images, following the standard 7:1:2 ratio.

The 14 pathologies in ChestX-ray14 are Atelectasis (Atel), Cardiomegaly (Card), Effusion (Effu), Infiltration (Infi), Mass, Nodule (Nodu), Pneumonia (Pneu1), Pneumothorax (Pneu2), Consolidation (Cons), Edema (Edem), Emphysema (Emph), Fibrosis (Fibr), Pleural Thickening (P_T) and Hernia (Hern), respectively.

The 14 pathologies in CheXpert, MIMIC-CXR, and IU X-Ray are No Finding (NoFi), Enlarged Cardiomediastinum (EnCa), Cardiomegaly (Card), Lung Lesion (Lesi), Lung Opacity (Opac), Edema (Edem), Consolidation (Cons), Pneumonia (Pneu1), Atelectasis (Atel), Pneumothorax (Pneu2), Pleural Effusion (Effu), Pleural Other (Other), Fracture (Frac) and Support Devices (Devi), respectively.

For the labels of datasets, we use a D-dimensional vector of the one-hot form *y* = [$y_1$, $y_2, \ldots , y_d$], where d represents the number of disease classes and y is calculated by (2). $y_i \in \{0, 1\}$ denotes the presence or non-presence of disease category i, where 1 represents the presence and 0 represents absence. If $y_1$, $y_2, \ldots$, and $y_d$ are all 0, it means that the above disease type does not exist in this image.

**Table 3:** Parameters for training.

Early stopping	True
Learning rate	0.0001
Patience	5
Batch size	64
Optimizer	Adam
Weight decay	1e−5
Batch size	64
Epochs	60
System configuration	Nvidia GTX 3060, 12G

### Implementation details

As shown in Table 3, we utilized the early stopping method with a patience of 5 to enhance training efficiency. We use the PyTorch framework to implement the MBRANet model. We trained the model on an NVIDIA GeForce RTX 3060 GPU with epochs of 60, and batch_size was set to 64. We first resized the original image to $256 \times 256$, then randomly cropped it to $224 \times 224$ and used a random horizontal flip operation, and normalized it using the mean and standard deviation of ImageNet^42^. We used the Adam^43^ optimizer with a weight decay of 1e−5, a beta of (0.9, 0.999), and a eps of 1e−8. The initial learning rate was set to 0.0001. When the loss value no longer decreases or the mean AUC value no longer increases over five epochs, the learning rate is divided by 10. In the validation and testing phases, we still resize the original image to $256 \times 256$, random crop to $224 \times 224$, and perform the same normalization operation as in the training phase.

### Evaluation metrics

Because of the class imbalance problem, the area under the receiver operating characteristic curve(AUC) is a more reasonable indicator for performance evaluation than other indicators (e.g. accuracy, F1-Score), which has been used in most relevant work. Following^28,44^, we used the per-class AUC score to measure the performance of these methods in diagnosing each of the 14 thoracic diseases. A high AUC value is known to indicate better performance of the model and higher diagnostic accuracy. Therefore, we will use the AUC metric to compare with other models. In the Receiver Operating Characteristic (ROC) curve, the horizontal axis is the False Positive Rate (FPR) and the vertical axis is the True Positive Rate (TPR), FPR and TPR are calculated through (8). By maintaining high TPR and low FPR across thresholds, a good ROC curve provides a comprehensive evaluation of the model’s discrimination power and overall performance in classification tasks. Additionally, we evaluated some models based on parameter count, FLOPS, GPU memory usage, training time, and inference time.

8 $$\begin{aligned} \begin{array}{l} {\text {FPR}} = \dfrac{{{\text {FP}}}}{{{\text {FP}} + {\text {TN}}}}\\ \\ {\text {TPR}} = \dfrac{{{\text {TP}}}}{{{\text {TP}} + {\text {FN}}}} \end{array} \end{aligned}$$

### Comparison with state-of-the-art methods

**Figure 7:**
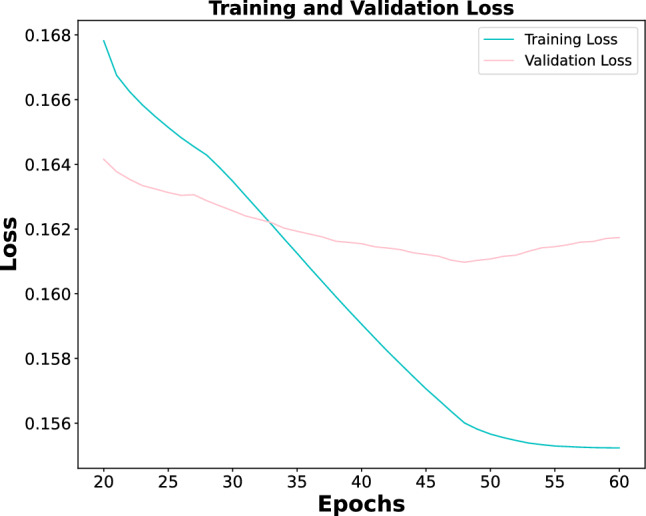
Illustration of the training and validation loss curves of the proposed MBRANet framework on the ChestX-Ray14 dataset.

**Figure 8:**
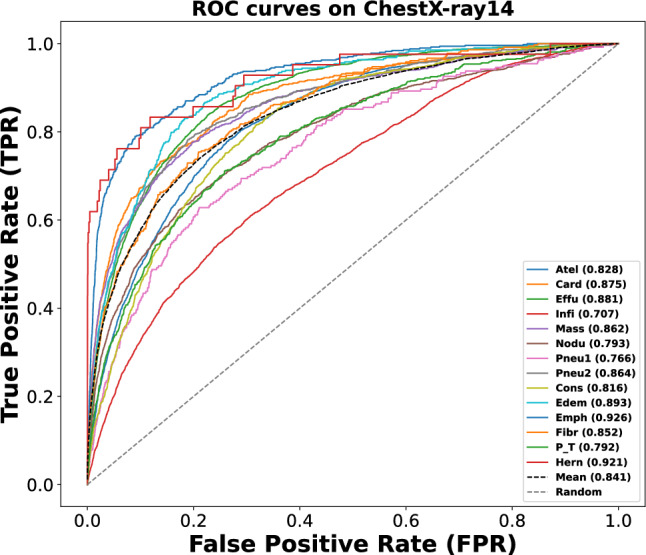
Illustration of ROC curves of the proposed MBRANet framework on the ChestX-Ray14 dataset.

In this section, we do extensive experiments and analysis on two benchmark public datasets, ChestX-Ray14, CheXpert, MIMIC-CXR, and IU X-Ray. We use the AUC metric to evaluate the final effect of MBRANet and compare it with current state-of-the-art methods. In Tables 4 and 5, we record not only the AUC score for each chest disease but also the mean AUC score values for all chest diseases.

**Table 4:** Comparison with previous baselines on the ChestX-Ray14 dataset. The AUC score of each pathology and the mean AUC score of 14 pathologies are reported. For each column, the best results are highlighted in bold.

SOTA	Atel	Card	Effu	Infi	Mass	Nodu	Pneu1	Pneu2	Cons	Edem	Emph	Fibr	P_T	Hern	Mean
Ho et al. (2019)^45^	0.795	0.887	0.875	0.703	0.835	0.716	0.742	0.863	0.786	0.892	0.875	0.756	0.774	0.836	0.810
Ouyang et al. (2020)^46^	0.770	0.870	0.830	0.710	0.830	0.790	0.720	0.880	0.740	0.840	0.940	0.830	0.790	0.910	0.819
Chen et al. (2020)^28^	0.786	0.893	0.832	0.699	0.840	0.800	0.739	0.876	0.751	0.850	0.944	0.834	0.795	0.929	0.826
Ho et al. (2020)^47^	0.794	0.896	0.882	0.705	0.844	0.752	0.763	0.878	0.798	0.870	0.918	0.803	0.779	0.909	0.822
Kim et al. (2021)^48^	0.780	0.887	0.835	0.710	0.831	0.804	0.734	0.871	0.747	0.840	0.941	0.815	0.779	0.909	0.822
Guan et al. (2021)^49^	0.785	0.889	0.835	0.699	0.838	0.775	0.738	0.871	0.763	0.850	0.924	0.831	0.776	0.922	0.822
Wang et al. (2021)^30^	0.779	0.895	0.836	0.710	0.834	0.777	0.737	0.878	0.759	0.855	0.933	0.838	0.791	**0.938**	0.826
Chen et al. (2021)^44^	0.792	0.892	0.840	0.714	0.848	**0.812**	0.733	0.885	0.753	0.848	**0.948**	0.827	0.795	0.932	0.830
Chen et al. (2022)^34^	0.807	0.910	0.879	0.698	0.824	0.750	0.750	0.850	0.802	0.888	0.890	0.812	0.768	0.915	0.825
Lin et al. (2023)^50^	0.790	**0.944**	0.882	0.715	0.846	0.768	0.714	0.850	0.728	**0.893**	0.883	0.798	0.762	0.908	0.820
Jiang et al. (2022)^51^	0.798	0.896	0.842	**0.719**	0.856	0.809	0.758	0.879	0.759	0.849	0.906	0.847	**0.800**	0.913	0.830
Taslimi et al. (2022)^52^	0.781	0.875	0.824	0.701	0.822	0.780	0.713	0.871	0.748	0.848	0.914	0.826	0.778	0.855	0.810
Wu et al. (2023)^53^	0.742	0.918	0.820	0.684	0.763	0.697	0.596	0.820	0.720	0.840	0.810	0.783	0.674	0.890	0.768
Öztürk et al. (2023)^54^	0.810	0.904	0.878	0.712	**0.874**	0.783	0.759	**0.894**	**0.822**	0.882	0.908	0.824	0.797	0.887	0.838
Sonit Singh (2024)^55^	0.816	0.911	**0.884**	0.708	0.831	0.775	0.765	0.874	0.801	0.898	0.923	0.829	0.786	0.901	0.835
Ours	**0.828**	0.875	0.881	0.707	0.862	0.793	**0.766**	0.864	0.816	0.893	0.926	**0.852**	0.792	0.921	**0.841**

#### Evaluation on ChestX-Ray14 dataset

First, Fig. 7 gives the tendency of the loss values during training and validation. Then, we evaluate the performance of our model on the test dataset of ChestX-Ray14. The AUC score values for each lung disease are summarized in Table 4, where our proposed MBRANet achieves an average AUC value of 0.841 in 14 lung diseases, which is better than the other State-of-the-art Methods in Table 4. Figure 8 shows the ROC curves of the proposed MBRANet for the 14 lung diseases on the test dataset.

According to the results in Table 4, our proposed MBRANet achieved the best performance in three diseases (Atelectasis, Pneumonia, and Fibrosis). It achieved the highest mean AUC value of 0.841 among all 14 diseases, which effectively demonstrates the effectiveness of our proposed MBRANet for multi-label classification. As Compared to the mean AUC values of 0.830 of the State-of-the-art Methods^44,51^ in Table 4, we improved by over 1%. Moreover, our research consistently outperforms transformer-based methods^52–55^, achieving an accuracy of 0.841 compared to their reported accuracies of 0.768, 0.838, and 0.835. This further demonstrates that our MBRANet is superior to other State-of-the-art Methods. It is worth noting that our method achieved the best results in Atel, pneu1, and Fibr diseases, increasing all three conditions by around 1% compared to the highest AUC values previously achieved in these three diseases. For Effu, Edema, P_T, Infi, and Hern diseases, the AUC values we obtained are very close to the best values obtained by state-of-the-art methods for these diseases. However, it is evident from the table that for Card, Nodu, pneu2, and Emph conditions, our method does not achieve comparable results. In the future, we will conduct targeted research on this issue.

Besides, among the 14 diseases, we found that the AUC score for “Infiltration” was very low among all methods, probably because the diagnosis of this condition relies mainly on minor textural changes in CXR images, and it is still a challenge to improve the recognition of this disease.

#### Evaluation on CheXpert dataset

This section reports the effect of MBRANet on the CheXpert dataset. First, we evaluate the performance of our model on a validation set in the official^3^. Since uncertain labels are present in the training set, we use two approaches to deal with uncertain labels, U-Ones and U-Zeros. The comparison results obtained by MBRANet with other State-of-the-art Methods are shown in Table 5.

As displayed in the experimental results in Table 5. When utilizing the U-Ones method for uncertain labels, it is evident that^55^ achieved the highest mean AUC value of 0.904 on the validation dataset with five categories.

**Table 5:** Comparison with previous baselines on the CheXpert dataset as measured by the AUC score of the validation set. The U-Ones and U-Zeros are different settings for uncertain labels. For each column, the best results are highlighted in bold.

SOTA	Method	Atel	Card	Cons	Edem	Effu	EnCa	Lesi	Opac	NoFi	Other	Pneu1	Pneu2	Devi	Frac	Mean
Irvin et al. (2019)^3^	U-Ones	0.858	0.832	0.899	**0.941**	0.934	–	–	–	–	–	–	–	–	–	0.893
Pham et al. (2021)^56^	U-Ones	0.800	0.780	0.882	0.918	0.920	–	–	–	–	–	–	–	–	–	0.860
Chen et al. (2021)^44^	U-Ones	0.728	**0.887**	0.728	0.890	0.915	0.678	0.795	0.809	0.889	0.813	**0.799**	**0.913**	**0.917**	**0.829**	0.829
Sonit Singh (2024)^55^	U-Ones	**0.862**	0.861	**0.916**	0.936	**0.944**	–	–	–	–	–	–	–	–	–	0.904
Sonit Singh (2024)^55^	U-Ones	0.713	0.825	0.738	0.883	0.887	0.658	0.829	0.805	**0.912**	**0.932**	0.735	0.838	0.891	0.737	0.813
Ours	U-Ones	0.858	0.851	0.896	0.937	0.933	–	–	–	–	–	–	–	–	–	0.895
Ours	U-Ones	0.860	0.850	0.896	0.938	0.931	**0.659**	0.803	**0.810**	0.842	0.852	0.702	0.841	0.893	0.730	0.829
Irvin et al. (2019)^3^	U-Zeros	0.811	**0.840**	**0.932**	0.929	0.931	–	–	–	–	–	–	–	–	–	0.886
Pham et al. (2021)^56^	U-Zeros	0.745	0.813	0.882	0.921	0.930	–	–	–	–	–	–	–	–	–	0.858
Chen et al. (2021)^44^	U-Zeros	0.747	0.882	0.785	0.888	0.916	**0.687**	0.797	0.811	**0.893**	0.814	**0.813**	**0.912**	**0.926**	**0.827**	0.836
Ours	U-Zeros	0.839	0.832	0.895	0.949	**0.940**	–	–	–	–	–	–	–	–	–	0.891
Ours	U-Zeros	**0.839**	0.836	0.893	**0.949**	0.938	0.663	**0.831**	**0.830**	0.855	**0.848**	0.711	0.855	0.921	0.730	0.835

**Table 6:** Comparison with previous baselines on the MIMIC-CXR and IU X-Ray datasets as measured by the AUC score of the test set. Significant values are in bold.

SOTA	Dataset	Atel	Card	Cons	Edem	Effu	EnCa	Lesi	Opac	NoFi	Other	Pneu1	Pneu2	Devi	Frac	Mean
Hou et al. (2021)^57^	MIMIC	0.722	0.730	0.728	0.799	0.863	0.767	0.623	0.653	0.817	0.588	0.797	0.789	0.917	0.560	0.721
Sonit Singh (2024)^55^	MIMIC	0.766	0.794	0.712	**0.841**	**0.898**	0.682	0.712	0.703	0.812	0.799	0.718	**0.803**	**0.913**	0.663	0.773
Ours	MIMIC	**0.779**	**0.825**	**0.788**	0.833	0.863	**0.823**	**0.735**	**0.788**	**0.835**	**0.821**	**0.823**	0.769	0.879	**0.703**	**0.805**
Sonit Singh (2024)^55^	IU	0.783	0.736	0.522	0.742	0.583	0.726	0.544	0.694	0.730	0.849	0.716	0.620	0.717	0.611	0.684
Ours	IU	**0.795**	**0.741**	**0.642**	**0.746**	**0.614**	**0.787**	**0.603**	**0.759**	**0.800**	**0.894**	**0.799**	**0.775**	**0.748**	**0.707**	**0.745**

Compared to the mean AUC for the five disorders in^3,55^, we obtained a mean value that was superior to^3,55^ (0.895 vs. 0.893, 860). When validated in the 14 classes, the MBRANet method achieves the same high AUC value as^44^, outperforming the AUC value of 0.813 in^55^. When we use the U-Zeros method for uncertain labels, as shown by the results in Table 5, the MBRANet method obtains better results (0.891 vs. 0.886, 0.858) for validation of the five categories. For the validation of 14 categories, we obtained an AUC value of 0.835, which is close to the value in^44^. We found that the U-Zeros method performs better than the U-Ones method. This phenomenon occurred because the number of noisy labels must have become less when we mapped the uncertain label to a new label, which gives a better training result.

#### Evaluation on MIMIC-CXR dataset

This section presents a detailed summary of the performance of the MBRANet model on the MIMIC-CXR dataset. The comparison in Table 6 demonstrates that our model outperformed^55,57^ with the highest Mean AUC value of 0.805, surpassing their values of 0.721 and 0.773. Notably, while^55^ achieved better AUC values for Edem, Effu, Pneu2, and Devi, our model obtained the highest AUC values for the remaining diseases. These results underscore the effectiveness and robustness of our model in disease classification tasks.

#### Evaluation on IU X-Ray dataset

According to the results in Table 6, our model achieved a mean AUC value of 0.745 across the 14 diseases in the IU X-Ray dataset. Even more excitingly, our model obtained higher AUC values for each disease compared to the results in^55^. However, combining the information from Tables 5 and 6, it is evident that our model has outperformed its performance in the IU X-Ray dataset on the same 14 diseases in both the CheXpert and MIMIC-CXR datasets.

The MBRANet model has been trained and evaluated on four publicly available datasets, namely ChestX-Ray14, CheXpert, MIMIC-CXR, and IU X-ray. The results of the evaluation demonstrate that MBRANet achieves state-of-the-art performance on all four datasets. This indicates that MBRANet has excellent generalization capability.

## Analysis and discussions

In this section, We first analyze the reasons for the effectiveness of our proposed MBRANet network. Next, we will use a Graphical User Interface (GUI) to display the final analysis results of our model more clearly. This allows us to perform visual analysis.

### Effectiveness analysis of the MBRANet

#### Ablation studies

**Table 7:** Ablation experiments on CA/MFC/BCEWITHLABELSMOOTHING modules. In this context, MFC refers to combining FPN and CSRA modules. The mean AUC scores are reported for 14 pathologies on the ChestX-Ray14 dataset.

CA	MFC	BCEWithLabelSmoothing	Mean	FLOPS(G)	PARAMS(M)	GPU memory(G)	Inference time(s)
✘	✘	✘	0.831	4.13	23.54	9.59	0.0268
✔	✘	✘	0.834	4.14	23.67	10.24	0.0281
✘	✔	✘	0.832	4.96	26.28	10.55	0.0273
✘	✘	✔	0.836	4.13	23.54	9.59	0.0268
✔	✔	✘	0.836	4.96	26.41	11.45	0.0289
✔	✔	✔	0.841	4.96	26.41	11.45	0.0289

**Table 8:** Computational cost comparison of different methods.

Method	FC	CSRA	SE	CBAM	CA
FLOPS(M)	0.0573	0.1294	2.0200	4.0000	5.0800
PARAMS(M)	0.0287	0.0135	0.6288	0.6303	0.1355
Mean	0.8360	0.8410	0.8310	0.8320	0.8410

Table 7 summarized the mean AUC scores for the 14 pathologies on the ChestX-Ray14 dataset using different modules. Additionally, it offers a detailed comparison of the computational costs associated with different models, encompassing parameters, FLOPS, GPU memory utilization, and inference time. The average AUC value was 0.831 while using ResNet50 for classifying chest diseases. To prove the effectiveness of the CA, MFC, and BCEWithLabelSmoothing modules, we integrated each of these modules separately into the original ResNet50. After conducting our analysis, we discovered that the implementation of the CA module resulted in an average AUC score increase of 0.3% over the baseline (0.834 vs. 0.831). This demonstrates the effectiveness of the CA module and its ability to enhance our overall results. When using MFC, and BCEWithLabelSmoothing respectively, the AUC scores improved by 0.1% (0.832 vs. 0.831) and 0.5% (0.836 vs. 0.831) over our baseline in turn. This highlights the effectiveness of the MFC and BCEWithLabelSmoothing modules that we have designed. When using the CA and MFC modules together, the result is 0.836, improving by 0.5%, which is better than both modules alone (0.836 vs. 0.834, 0.832). In addition, when using all three modules together we achieved the best AUC score of 0.841. The results in Table 7 effectively demonstrate the effectiveness of each module.

Based on the cost comparison summarized in Table 8, it is advisable to opt for the CSRA module over the FC module. The CSRA module exhibits superior performance in terms of computational complexity and parameter count, making it the more efficient choice for the given scenario. Furthermore, selecting the CA module instead of the SE and CBAM modules aligns better with the overall analysis.

#### Analysis of feature extractor

**Table 9:** Effect of different backbone structures on the ChestX-Ray14 dataset. Training time refers to the time taken for one epoch. Significant values are in bold.

Method	FLOPS(G)	PARAMS(M)	GPU memory	Training time	Inference time	Mean
ConvNet	4.46	27.82	**2.76**	11 min 20 s	0.0265	0.716
SKNet	5.11	29.53	4.77	12 min 30 s	0.0271	0.815
DenseNet121	3.90	26.97	11.25	12 min 56 s	0.0301	0.830
ResNet18	**2.39**	**14.81**	7.91	**11 min 11 s**	**0.0232**	0.832
ResNet101	7.86	42.53	11.64	13 min 54 s	0.0412	0.830
ResNet50	4.96	26.41	10.45	12 min 20 s	0.0289	**0.841**

**Table 10:** Effect of different attention mechanisms for mean AUC Results on the ChestX-Ray14 dataset. Significant values are in bold.

Attention	Atel	Card	Effu	Infi	Mass	Nodu	pneu1	pneu2	Cons	Edem	Emph	Fibr	P_T	Hern	Mean
SE	0.819	0.869	0.866	0.692	0.850	0.785	0.761	0.840	0.795	0.881	0.916	0.854	0.774	0.925	0.831
CBAM	0.820	0.870	0.867	0.692	0.845	0.778	**0.767**	0.838	0.789	0.884	0.903	0.846	0.776	**0.967**	0.832
CA	**0.830**	**0.877**	**0.881**	**0.705**	**0.860**	**0.792**	0.766	**0.866**	**0.816**	**0.891**	**0.927**	**0.847**	**0.789**	0.922	**0.841**

The MBRANet model utilizes a fine-tuned ResNet50 as the backbone network for feature extraction. We conducted experiments to assess the efficacy of utilizing ResNet50 as the feature extractor. And we replaced ResNet50 with variations such as DenseNet121, ResNet18, SKNet, ConvNet, and ResNet101 models while keeping all other settings constant. To improve the learning efficiency of the model, the backbone network used in the experiments will be the corresponding version of the pre-trained model on the ImageNet dataset. Table 9 presents a summary of the experimental results, showing that the best results were achieved using ResNet50 on the ChestX-Ray14 dataset with a mean AUC score of 14 pathologies (0.841).

In deep learning models, factors such as the number of parameters, network depth, and architecture play a crucial role in determining model performance. Analysis of Table 9 results indicates that ResNet50, with its moderate parameter count and network depth, exhibits stronger feature learning and representation capabilities. This translates to higher accuracy, superior generalization, and a more efficient and stable training process. Despite its superiority in FLOPS, PARAMS, and training time, ResNet18 only achieves a Mean AUC of 0.832. Consequently, ResNet50 outperforms ResNet101 and other models by adapting better to datasets and achieving superior performance, making it a preferred choice for various deep learning applications.

#### Analysis of attention mechanisms

The use of attention mechanisms has a significant impact on the experimental results. After we chose ResNet50 as the backbone network of MBRANet, we introduced different attention mechanism approaches into the residual blocks. Different attention mechanisms were introduced at positions after 3 $\times$ 3 convolution in the residual block. Table 10 summarises the results obtained on the ChestX-Ray14 test dataset using the SE, CBAM, and CA attention mechanism methods that employ the MFC and BCEWithLabelSmoothing techniques. The structure is shown in Fig. 3. The best results were obtained using the CA approach (0.841 vs. 0.830, 0.831). Based on the results in Tables 8 and 10, the CA attention mechanism approach was chosen to help ResNet50 process the image data better, to improve the performance and accuracy of the model.

For a given input, the CA module encodes each channel along the horizontal and vertical directions, producing a set of direction-aware feature maps. The operation captures correlations between pixels over long distances and retains positional information as a way to help localize our network to the region of interest.

**Table 11:** Multi-scale feature aggregation method comparison.

Method	Add	Concat	MFC
FLOPS(G)	2.8188	2.8204	2.0211
PARAMS(M)	2.8647	3.0719	0.0251
Mean	0.8370	0.8330	0.8410

#### Analysis of multi-branch feature classifier (MFC)

Table 11 shows that the MFC module excels over the Concat and Add methods in multiple aspects. The MFC module has the lowest computational complexity at 2.0211G, while the Add and Concat methods have higher values at 2.8188G and 2.8204G, respectively. In terms of parameter size, the MFC module is the smallest at 0.0251M, contrasting with the larger sizes of 2.8647M for Add and 3.0719M for Concat. Additionally, the MFC module achieves the highest Mean AUC score of 0.8410, outperforming the Add at 0.8370 and the Concat at 0.8330. Hence, the MFC module is the preferred choice due to its superior computational efficiency, smaller parameter size, and better model performance compared to the Add and Concat methods.

#### Analysis of $\lambda$-parameters

In the MFC method we designed, the only hyperparameter is $\lambda$. We take to evaluate the performance of different $\lambda$ on the ChestX-Ray14 datasets. Figure 5 illustrates the CSRA module in the MFC method. $\lambda$ controls the effect of the spatial pooling component, but when $\lambda$ is too large, the contribution of the average pooling component is diminishing. Therefore, for multi-scale features, the appropriate size of $\lambda$ to improve the classification results is very important. For finding a suitable setting of $\lambda$, we kept the other settings unchanged in the experiment and evaluated the impact of different sets of $\lambda$ values ($\lambda _1, \lambda _2, \lambda _3, \lambda _4$) on the final classification results. $\lambda _1, \lambda _2, \lambda _3, \lambda _4$ correspond to the $\lambda$ parameter value settings in the CSRA1, CSRA2, CSRA3 and CSRA4 modules in Fig. 2, respectively. Table 12 summarizes the experimental results for 10 different sets of $\lambda$ values. Table 12 shows that this set of $\lambda$ values of (0.5, 0.4, 0.3, 0.1) achieved the best mean AUC value (0.841). Notably, in future work, we can further improve classification performance by setting more groups of $\lambda$ values.

**Table 12:** Effect of setting different $\lambda$-parameters for mean AUC results on the ChestX-Ray14 dataset. Significant values are in bold.

$\lambda$-parameters	Atel	Card	Effu	Infi	Mass	Nodu	pneu1	pneu2	Cons	Edem	Emph	Fibr	P_T	Hern	Mean
(0.1, 0.1, 0.1, 0.1)	0.821	0.871	0.869	0.691	0.849	0.776	0.764	0.843	0.801	0.887	0.901	**0.850**	0.784	0.936	0.832
(0.2, 0.2, 0.2, 0.2)	0.824	0.863	0.873	0.695	0.856	0.787	0.763	0.844	0.801	0.878	0.901	0.846	0.788	0.938	0.833
(0.3, 0.3, 0.3, 0.3)	0.823	0.874	0.872	0.696	0.850	0.786	0.768	0.847	0.803	0.884	0.911	0.846	0.783	0.943	0.834
(0.4, 0.4, 0.4, 0.4)	0.824	0.874	0.872	0.698	0.854	0.787	0.770	0.852	0.806	0.884	0.917	0.849	**0.794**	**0.944**	0.837
(0.5, 0.5, 0.5, 0.5)	0.824	**0.878**	0.873	0.697	0.857	0.783	**0.775**	0.852	0.805	0.887	0.914	0.846	0.786	0.927	0.836
(0.5, 0.3, 0.2, 0.1)	0.820	0.869	0.868	0.696	0.854	0.786	0.769	0.844	0.799	0.885	0.910	0.849	0.781	0.912	0.831
(0.5, 0.3, 0.2, 0.4)	0.818	0.870	0.867	0.688	0.850	0.781	0.767	0.839	0.792	**0.893**	0.904	0.848	0.792	0.943	0.833
(0.4, 0.3, 0.2, 0.1)	0.820	0.868	0.868	0.690	0.850	0.782	0.767	0.844	0.799	0.880	0.895	0.848	0.775	0.938	0.830
(0.5, 0.4, 0.3, 0.1)	**0.830**	0.877	**0.881**	**0.705**	**0.860**	**0.792**	0.766	**0.866**	**0.816**	0.891	**0.927**	0.847	0.789	0.922	**0.841**

### Problem analysis

**Figure 9:**
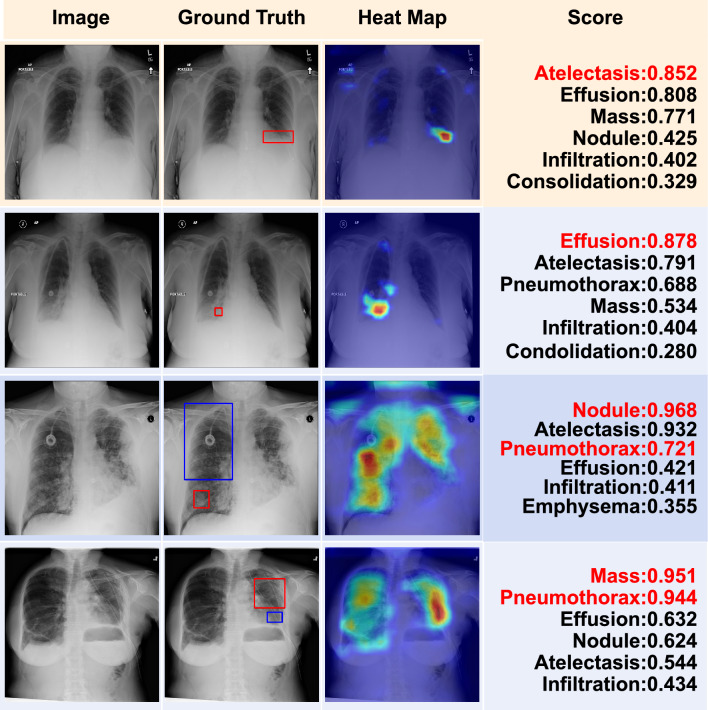
Localization of lesion regions with the proposed MBRANet model. The first column shows the raw images from the ChestX-Ray14 dataset. The second column is the ground truth, the manual lesion regions provided by the official version are annotated with bounding boxes. Note that we do not use any bounding boxes for training or testing. The third column shows the heat maps for the corresponding CXR examples. The higher response is represented by the color red, and the lower response is represented by the color blue. The last column shows the test results of multi-label CXR image classification with MBRANet. The top 6 predicted categories and their corresponding probability scores are presented. The ground truth pathologies are highlighted in red.

When classifying thoracic diseases, to solve the problem of localizing lesion regions in different sizes, we introduce CA in the residual block to suppress the noise interference in the CXR image and capture the correlation between distant pixels to help the network localize to the region of the lesion. In most of the existing research methods, the extracted multi-scale features are not capitalized to improve the performance of the model. In addition, there is a severe sample imbalance in certain pathologies within the dataset, which tends to create an overdependence on a limited number of samples leading to overfitting. To address the above issues, we designed the MFC method to handle multi-scale information features and use decision fusion to produce the final classification results. In addition, we employed the label smoothing technique to address the issue of sample imbalance by processing the one-hot labels of the images.

We do the training and validation on the ChestX-Ray14, CheX-pert, MIMIC-CXR, and IU X-Ray datasets. In Tables 4, 5, and 6, we compared our results with previous studies on the same datasets, with the AUC value serving as the main performance metric. The analysis revealed that the proposed models are highly efficient and boast excellent generalization ability. It is important to note that while some studies have shown consistent findings, others have reported inconsistent results, underscoring the importance of meticulous data analysis to ensure accuracy and reliability.

**Figure 10:**
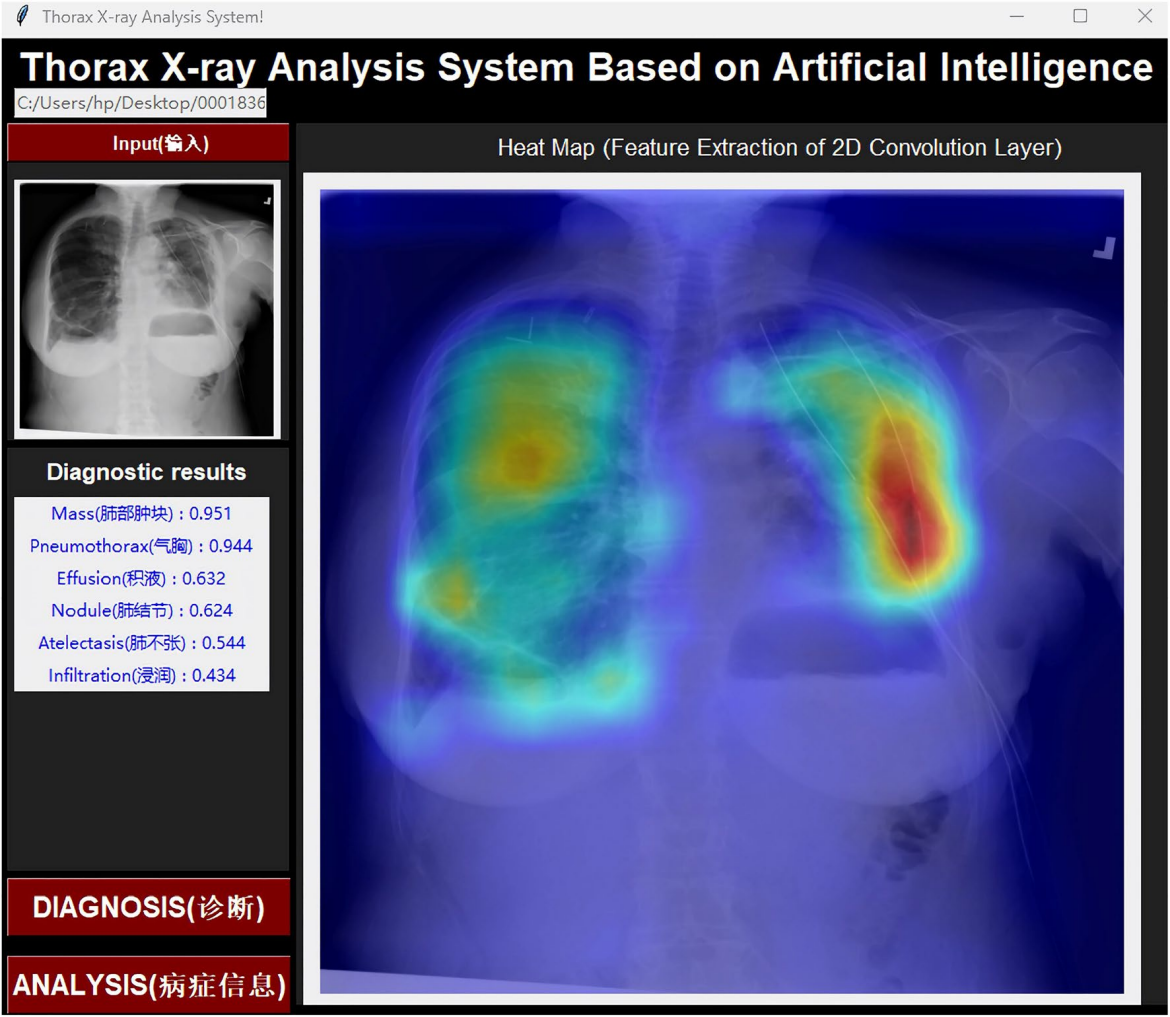
The interface of the Thorax X-ray Analysis System. First, we upload the CXR image by clicking on the INPUT button. Next, by clicking on the DIAGNOSIS button, MBRANet is called to diagnose the CXR image, and the top 6 results are displayed on the page, and the heat map generated by Grad-CAM is also displayed. In addition, clicking on the ANALYSIS button will display information about the disease.

### Case analysis

With the advantage of weakly supervised localization, we use Grad-CAM^58^ to generate a heatmap of the CXR image to highlight the lesion region. As shown in Fig. 9, the highlighted areas on the heatmap closely resemble Ground Truth, demonstrating that our proposed method can locate the most discriminative lesion areas on CXR images. In addition, we show the predicted results of multi-label classification on the ChestX-Ray14 dataset. In Fig. 9, the score column shows the top 6 predicted scores of MBRANet for each sample in predicting thoracic diseases. It indicates that our proposed method obtains accurate classification results.

To apply the trained MBRANet to assist in the diagnosis of thoracic diseases, we used Tkinter, a standard GUI (graphical user interface) toolkit for Python, to implement the visual case analysis. Figure 10 shows the main interface of the thorax X-ray analysis system implemented using Tkinter. The use of a GUI makes diagnostic results more intuitive and helps the medical professional diagnose the patient’s disease.

## Discussion

Most existing methods for classifying chest X-ray images face challenges in accurately capturing lesion details at different scales due to the lack of consideration for spatial and channel information within disease regions. Additionally, the common problem of class imbalance in datasets can lead to overfitting and reduce the accuracy of classification results. The absence of effective solutions further hinders the achievement of precise classification. To solve these issues, we propose a multi-branch residual attention network (MBRANet) model, which focuses on the fusion and classification of image features at several different scales.

When classifying thoracic diseases, to solve the problem of localizing lesion regions in different sizes, we introduce CA in the residual block to suppress the noise interference in the CXR image and capture the correlation between distant pixels to help the network localize to the region of the lesion. In most of the existing research methods, the extracted multi-scale features are not capitalized to improve the performance of the model. We also performed multi-scale feature fusion using FPN models. To tackle the issue of an excessive number of parameters in the FC layer, we designed the MFC method to handle multi-scale information features and use decision fusion to produce the final classification results. In addition, we use the labelSmoothing technique to process the one-hot labels of the images to solve the sample imbalance problem.

Our research involved training, validating, and testing our proposed MBRANet model on the ChestX-Ray14, CheXpert, MIMIC-CXR, and IU X-Ray datasets. In Tables 4, 5, and 6, we compared our results with previous studies on the same datasets, with the AUC value serving as the main performance metric. In addition, we also analyzed each module. These results indicate that our proposed model is highly efficient and boasts excellent generalization ability. It is important to note that while some studies have shown consistent findings, others have reported inconsistent results, underscoring the importance of meticulous data analysis to ensure accuracy and reliability.

Although the high classification performance of MBRANet has been demonstrated, there are still some limitations. In particular, when dealing with the relationship between different diseases, some diseases may not be predicted due to the possible overlap of lesion regions in different pathologies. In addition, as the labels of the dataset are encoded using one-hot coding, similar features may become independent after encoding. This may result in the loss of some useful information and make it difficult to process similar features. Please note that the classification results of diseases using MBRANet can only be used as a diagnostic aid.

Responding to these issues, our future work will further explore the potential correlations between different diseases. Integrating prior knowledge with our model across multiple modalities to improve the overall performance. We will also attempt to address the uncertainties associated with the use of one-hot labels.

## Conclusion

Experienced physicians possess the expertise to make accurate clinical diagnoses by focusing on the pathological information present in chest X-ray (CXR) images. In this paper, we proposed the MBRANet network model that can automatically learn multi-scale features of CXR images to classify some common chest diseases. In addition, we implement a GUI to call the trained MBRANet for computer-aided diagnosis. We trained and validated the performance of the models on the ChestX-Ray14, CheXpert, MIMIC-CXR, and IU X-Ray datasets. Our proposed MBRANet has the following features: (1) It can solve the problem of the inability of CNN to capture the long-range dependency of pixels by the Coordinate Attention (CA) module, Which enables our network to capture cross-channel information and direction-aware information. (2) It can accurately extract multi-scale image information and perform feature fusion, which improves the perceptual range of the network and improves the classification accuracy. (3) By replacing the fully connected (FC) layer with the class-specific residual attention (CSRA) module, we can not only reduce the number of parameters in the model but also improve the classification accuracy. (4) Using our designed BCEWithLabelSmoothing loss function to solve the problem of category imbalance in the dataset, which can reduce the overfitting tendency of the model. For future work, we will further explore the dependency between the information on disease characteristics to discriminate between the correct and incorrect categories with a blurred line.

## Data Availability

We clarify that our research findings are based on the analysis of four publicly available datasets: the ChestX-ray 14, CheXpert, MIMIC-CXR, and the IU X-Ray dataset. ChestX-ray 14: https://nihcc.app.box.com/v/ChestXray-NIHCC. CheXpert: https://stanfordmlgroup.github.io/competitions/chexpert. MIMIC-CXR: https://physionet.org/content/mimic-cxr/2.0.0/. IU X-Ray: https://openi.nlm.nih.gov/.
